# Thoracic Malignancies: From Prevention and Diagnosis to Late Stages

**DOI:** 10.3390/life15020138

**Published:** 2025-01-21

**Authors:** Julien Ancel, Laura Bergantini, Paolo Mendogni, Zhiwei Hu

**Affiliations:** 1 Université de Reims Champagne Ardenne, Inserm, UMR-S 1250 P3Cell, SFR CAP Santé, 51092 Reims, France; 2 CHU de Reims, Hopital Maison-Blanche, Service de Pneumologie, 51092 Reims, France; 3 Respiratory Diseases Unit, University of Siena, 53100 Siena, Italy; laurabergantini@gmail.com; 4 Thoracic Surgery and Lung Transplant Unit, Foundation IRCCS Ca’ Granda Ospedale Maggiore Policlinico, 20122 Milan, Italy; p.mendogni@gmail.com; 5 Pelotonia Institute for Immuno-Oncology, The Arthur G. James Comprehensive Cancer Center, College of Medicine, The Ohio State University Wexner Medical Center, Columbus, OH 43210, USA

Lung cancer remains the leading cause of cancer-related mortality worldwide, characterized by its complexity and heterogeneity. This Special Issue addresses pivotal aspects of lung cancer care, encompassing risk stratification, diagnostic advancements, tumor classification, therapeutic strategies, and the management of treatment-related complications. The contributions collectively highlight the ongoing evolution toward personalized and multidisciplinary care.

1. Understanding Cancer Risk and Enhancing Early Detection

Randomized controlled trials, such as the National Lung Screening Trial (NLST) [1] and the NELSON trial [2], have demonstrated that low-dose computed tomography (LDCT) screening reduces lung cancer mortality compared to chest radiography or no screening. While these findings are pivotal, gaps remain in optimizing screening strategies to ensure their clinical use and cost-effectiveness [3].

A critical limitation of current screening approaches is their primary focus on heavy smokers, potentially overlooking other at-risk populations [4]. It is increasingly evident that a significant proportion of lung cancers occur in non-smokers and light smokers. Therefore, identifying populations at risk of developing lung cancer beyond traditional smoking criteria is essential to design inclusive and effective screening programs.

Further advancements in risk stratification models are needed to better characterize individuals at elevated risk, regardless of smoking history [5]. This includes leveraging novel biomarkers, integrating chronic disease assessments (e.g., for COPD or cardiovascular disease), and utilizing artificial intelligence for lung nodule detection and risk prediction [6]. Expanding research into these areas could enable the development of tailored screening strategies for underrepresented groups, ensuring that non-smokers and light smokers are not excluded from the potential benefits of early detection.

Ultimately, addressing these gaps is crucial for creating equitable and efficient lung cancer screening programs that reflect the full spectrum of lung cancer risk.

The relationship between chronic lung diseases and lung cancer risk is a critical area of investigation. A systematic review presented in this Issue demonstrates that non-cystic fibrosis bronchiectasis (NCFB) is associated with a higher risk of lung cancer, particularly among elderly males and smokers [7]. These findings underline the importance of vigilance and targeted screening strategies in high-risk populations to facilitate early diagnosis and improve outcomes.

2. Advances in Diagnosis: Endoscopy and Beyond

The increasing adoption of LDCT screening programs has led to a growing number of detected radiological abnormalities, including pulmonary nodules [8]. These abnormalities are often smaller in size and identified at earlier stages, presenting unique diagnostic challenges [9,10]. As screening evolves, clinicians are faced with the dual tasks of distinguishing malignant lesions from benign ones and ensuring accurate, minimally invasive diagnostic approaches regarding these often-subtle findings.

To address these challenges, diagnostic technologies have advanced significantly, particularly in the field of endoscopy. Peripheral pulmonary lesions (PPLs), which are frequently detected through screening, represent a diagnostic frontier where conventional methods often fall short. Modern innovations such as robotic-assisted bronchoscopy, ultrathin bronchoscopes, and advanced navigation systems, including electromagnetic navigation and cone-beam CT, have dramatically improved diagnostic accuracy [11]. These tools enable the precise sampling of small or difficult-to-access lesions while minimizing invasiveness.

The integration of imaging-guided technologies further bridges the gap between radiology and interventional pulmonology. For instance, radial-probe endobronchial ultrasound (RP-EBUS) [12] and shape-sensing navigation systems allow clinicians to navigate complex bronchial pathways to access peripheral nodules, ensuring effective biopsy [13] and, in some cases, therapeutic intervention. Additionally, the ability to perform ablation or other localized treatments during diagnostic procedures underscores the evolving role of endoscopy in comprehensive lung cancer care.

Diagnostic innovations are reshaping lung cancer care, particularly in the evaluation of peripheral pulmonary lesions (PPLs). A detailed review of endoscopic technologies highlights advancements such as robotic bronchoscopy, ultrathin bronchoscopes, and navigation systems like cone-beam CT, which are revolutionizing diagnostic accuracy and enabling therapeutic interventions for PPLs [14]. These tools reflect a broader trend of integrating precision technologies into routine clinical practice, bridging the gap between early detection and effective treatment.

3. Deciphering Lung Adenocarcinoma Subtypes and Rare Tumors

Beyond advancements in diagnostic technologies and medical techniques, there have been significant breakthroughs in the understanding of the biology of thoracic cancers. These developments have led to a more nuanced classification of lung cancers, particularly lung adenocarcinomas, enabling a deeper insight into their histological subtypes [15,16]. This evolving knowledge, driven by molecular profiling and next-generation sequencing (NGS), has significantly altered the landscape of lung cancer classification.

The growing understanding of lung cancer heterogeneity is exemplified in a comprehensive review of rare lung adenocarcinoma subtypes [17], such as enteric and colloid types. These rare forms, once difficult to categorize, are now being studied in greater detail, with NGS providing insights into their unique molecular characteristics [18]. This progress paves the way for the development of more targeted therapeutic strategies, offering hope for improved outcomes in patients with these less common subtypes.

4. Evolving Therapeutic Strategies: From Stage III to Immunotherapy

The landscape of lung cancer treatment has evolved significantly over the years. Initially, therapy was centered around chemotherapy for all patients, often accompanied by radiotherapy, depending on the specific clinical scenario. However, treatment strategies have since undergone a profound transformation [19]. The development of novel drugs and therapeutic molecules has ushered in a new era of precision medicine, with targeted therapies emerging for cancers driven by oncogenic addictions [20]. Similarly, immunotherapy, particularly immune checkpoint inhibitors (ICIs), has revolutionized the treatment of advanced lung cancer.

Moreover, treatment regimens have become increasingly complex, with combinatory approaches now employed across various stages of lung cancer, including perioperative settings. This shift reflects a broader trend towards personalized and multidisciplinary care, optimizing treatment outcomes for patients at all stages of the disease [21].

However, despite these advances, therapeutic escalation is not always beneficial, and some patients may not respond to certain treatments. This highlights a critical need for predictive markers that can identify which patients will benefit from specific therapeutic strategies [22]. The ability to predict treatment efficacy is essential to avoid unnecessary side effects and to ensure that patients receive the most appropriate and effective therapies.

For stage III non-small cell lung cancer (NSCLC), the combination of induction therapy, surgery, and adjuvant strategies plays a crucial role. A study in this Issue identifies persistent nodal disease and lymph node ratio as strong prognostic indicators [23], emphasizing the importance of a thorough nodal assessment. These findings underscore the potential benefits of adjuvant therapy in improving long-term survival for these patients.

The role of immune checkpoint inhibitors (ICIs) in advanced NSCLC is also explored in this issue [24], with a particular focus on predictive biomarkers derived from PET/CT imaging. While GLCM-entropy was not predictive of treatment response in this study, the research demonstrates the feasibility of integrating imaging-derived texture features into routine clinical practice, providing a foundation for larger-scale investigations aimed at refining predictive biomarkers for better treatment stratification.

5. Managing Treatment-Related Toxicities and Monitoring Disease

In recent years, despite the therapeutic advances in lung cancer treatment, the risk of treatment-related toxicities remains a challenge. As new treatments, particularly immune checkpoint inhibitors (ICIs), become more prevalent, it is crucial to acknowledge the potential for a wide array of side effects [25]. Immunotherapy based on anti-PD-1/PD-L1 and CTLA-4 can cause toxicities that are often polymorphic and systemic, making the early recognition of symptoms essential for timely intervention [26]. These toxicities, which can affect various organs and systems, particularly the lung, necessitate a comprehensive understanding and vigilant monitoring to avoid potentially life-threatening complications.

As immunotherapy becomes a cornerstone of lung cancer treatment, the management of immune-related toxicities is of critical importance. A multicentre study in this issue examines immune checkpoint inhibitor-induced lung toxicity [27], revealing diverse clinical and radiological manifestations. These findings underscore the need for long-term monitoring and prompt intervention to mitigate severe complications and improve patient outcomes. This highlights the importance of not only recognizing these side effects early on but also adopting strategies for their effective management to reduce the risk of permanent damage or fatal outcomes [28].

In parallel, liquid biopsy emerges as a promising tool, offering a non-invasive method for monitoring treatment response and detecting early signs of progression [29]. The standard diagnostic procedure for non-small-cell lung cancer (NSCLC) typically involves the pathological evaluation of tissue samples obtained through surgery or biopsy, which are invasive and make repeat sampling a challenge, especially when progression occurs. Liquid biopsy circumvents this limitation by analyzing small fragments of circulating-free DNA (cfDNA), circulating tumor cells (CTCs), and cell-free RNA (cfRNA) shed from tumor cells into the bloodstream [30]. This non-invasive method enables a longitudinal assessment of genetic alterations in “druggable” genes, improving treatment follow-up and allowing for the earlier detection of progression compared to traditional imaging techniques, such as computed tomography (CT).

Although its sensitivity remains a challenge, liquid biopsy provides significant advantages, particularly in its ability to serve as a diagnostic, prognostic, and monitoring tool for patients undergoing molecularly targeted therapies or immunotherapy. These aspects have been reviewed in an article in this Special Issue [31]. As technological advancements continue to improve the precision and sensitivity of liquid biopsies, they are poised to become a standard part of lung cancer care. This technique could provide valuable insights into tumor biology, be used to track minimal residual disease, and offer the possibility of the early detection of treatment-related complications. Despite its current limitations, such as its lower sensitivity in some cases, the growing body of research and its potential applications in monitoring metastatic spread and personalizing treatment regimens indicate that liquid biopsy holds great promise. As such, liquid biopsy represents an exciting avenue for enhancing the management and monitoring of lung cancer, complementing traditional diagnostic methods, and transforming the approach to personalized treatment strategies.

6. Conclusions

This Special Issue illustrates the multidimensional nature of progress in lung cancer care, from risk stratification and diagnostic innovation to personalized therapy and toxicity management. By addressing these challenges holistically, the field continues to move closer to achieving precision medicine, improving survival and quality of life for patients worldwide.

## Data Availability

No new data were created in this editorial article. Data are available in the cited references.
